# Wide Distribution of O157-Antigen Biosynthesis Gene Clusters in *Escherichia coli*

**DOI:** 10.1371/journal.pone.0023250

**Published:** 2011-08-18

**Authors:** Atsushi Iguchi, Hiroki Shirai, Kazuko Seto, Tadasuke Ooka, Yoshitoshi Ogura, Tetsuya Hayashi, Kayo Osawa, Ro Osawa

**Affiliations:** 1 Interdisciplinary Research Organization, University of Miyazaki, Miyazaki, Japan; 2 Department of International Health, Graduate School of Health Sciences, Kobe University, Kobe, Japan; 3 Division of Bacteriology, Osaka Prefectural Institute of Public Health, Osaka, Japan; 4 Division of Microbiology, Department of Infectious Diseases, Faculty of Medicine, University of Miyazaki, Miyazaki, Japan; 5 Division of Bioenvironmental Science, Frontier Science Research Center, University of Miyazaki, Miyazaki, Japan; 6 Department of Bioresource Science, Graduate School of Agricultural Science, Kobe University, Hyogo, Japan; Charité-University Medicine Berlin, Germany

## Abstract

Most *Escherichia coli* O157-serogroup strains are classified as enterohemorrhagic *E. coli* (EHEC), which is known as an important food-borne pathogen for humans. They usually produce Shiga toxin (Stx) 1 and/or Stx2, and express H7-flagella antigen (or nonmotile). However, O157 strains that do not produce Stxs and express H antigens different from H7 are sometimes isolated from clinical and other sources. Multilocus sequence analysis revealed that these 21 O157:non-H7 strains tested in this study belong to multiple evolutionary lineages different from that of EHEC O157:H7 strains, suggesting a wide distribution of the gene set encoding the O157-antigen biosynthesis in multiple lineages. To gain insight into the gene organization and the sequence similarity of the O157-antigen biosynthesis gene clusters, we conducted genomic comparisons of the chromosomal regions (about 59 kb in each strain) covering the O-antigen gene cluster and its flanking regions between six O157:H7/non-H7 strains. Gene organization of the O157-antigen gene cluster was identical among O157:H7/non-H7 strains, but was divided into two distinct types at the nucleotide sequence level. Interestingly, distribution of the two types did not clearly follow the evolutionary lineages of the strains, suggesting that horizontal gene transfer of both types of O157-antigen gene clusters has occurred independently among *E. coli* strains. Additionally, detailed sequence comparison revealed that some positions of the repetitive extragenic palindromic (REP) sequences in the regions flanking the O-antigen gene clusters were coincident with possible recombination points. From these results, we conclude that the horizontal transfer of the O157-antigen gene clusters induced the emergence of multiple O157 lineages within *E. coli* and speculate that REP sequences may involve one of the driving forces for exchange and evolution of O-antigen loci.

## Introduction

The O antigen constitutes the outermost part of the lipopolysaccharide (LPS) present in the outer membrane of Gram-negative bacteria. The chemical composition and structure of O antigen exhibit high levels of variation even within a species, and the serotyping of strains with O antigens (together with the H-flagellar antigen) is used as an effective method to identify various pathogenic clones. In *Escherichia coli*, more than 170 O serogroups have so far been identified [1], and above all, the O157 is an important *E. coli* O serogroup because it is the most frequently reported O serogroup of enterohemorrhagic *E. coli* (EHEC) strains associated with outbreaks and sporadic cases of diarrhea, hemorrhagic colitis and hemolytic-uremic syndrome worldwide [2].

O157 strains isolated from patients with diarrhea usually carry EHEC-associated virulence genes, such as *stx1* and/or *stx2* (encoding Shiga toxins) and *eae* (encoding intimin). Additionally, the expression of the H7 antigen (encoded by *fliC*_H7_) is also an important characteristic of EHEC O157. However, some O157 strains do not carry *stx* genes, and express H antigens different from H7. These O157:non-H7 serotype strains are sometimes isolated from human and other sources worldwide [3], [4], [5], [6], [7]. O157:H45 strains have been isolated from diarrhea patients [5]. They possess both the *eae* and *bfpA* genes (encoding a subunit of bundle-forming pili), and were classified into a typical enteropathogenic *E. coli* (EPEC). O157:H39 strains carrying the *eae* gene were also isolated from diarrhea or asymptomatic cases [3], [6]. O157:H16 strains have occasionally been isolated from clinical, food or environmental sources, and some of these strains also carry the *eae* gene [3]. In addition to *eae*-positive strains, the presence of *eae*-negative O157:non-H7 strains (including O157:H10, O157:H16 and O157:H43) has also been reported [6].

In *E. coli*, genes required for O-antigen biosynthesis are usually clustered at a chromosomal locus flanked by the colanic acid biosynthesis gene cluster (*wca* genes) and the histidine biosynthesis (*his*) operon. And in EHEC O157, 12 genes required for the O157-antigen synthesis are clustered; *rfbE*, *gmd*, *fel*, *wbdQ*, *manC* and *manB* are involved in nucleotide sugar biosynthesis, *wbdN*, *wbdO*, *wbdP* and *wbdR* in sugar transfer (encoding glycosyl transferases), and *wzy* (encoding an O-antigen polymerase) and *wzx* (encoding a flippase) in O-antigen processing [8]. Recently, Feng *et al*. [3] demonstrated that *eae*-positive O157:non-H7 strains belonged to distinct evolutionary lineages from that of EHEC O157:H7 strains, suggesting a wide distribution of O157-antigen biosynthesis gene clusters within *E. coli*. However little is known about the characteristics of the O157-antigen gene cluster of O157:non-H7 strains.

Here, we examined 21 O157:non-H7 strains in order to study the evolution of the *E. coli* O157-serogroup strains. Sequence comparison with EHEC O157:H7 strains revealed that O157-antigen gene clusters are highly conserved among the strains, but can be divided into two distinct types at the nucleotide sequence level. Distribution of the two types did not clearly follow the evolutionary lineages of the strains, suggesting that horizontal transfer of the two distinct O157-antigen gene clusters induced the emergence of multiple O157 lineages within *E. coli*. Additionally, the observation suggests that horizontal transfer of O157-antigen gene cluster may be a prominent mechanism for the exchange of O-antigen loci. We discuss the probable mechanisms involved in the recombination of the fragments including O-antigen gene clusters.

## Materials and Methods

### Ethics Statement

An ethics statement is not required for this study according to the ethical guidelines for Epidemiological Studies of the Ministry of Health, Labor and Welfare, Japan.

### Bacterial Strains

The bacterial strains used in this study are listed in Table 1. All O157:non-H7 strains were isolated from human stool samples (except a strain, PV06-4 which was isolated from a food source) through routine investigations for outbreaks or sporadic cases of EHEC O157 during 1995–2006 in the Osaka Prefectural Institute of Public Health, Japan. An informed consent from patient involved is not applicable for this study, because the samples were taken for diagnostic purposes in order to appropriately treat the patients. Three O157:H7 strains: RIMD 0509952 referred to as Sakai [9], ATCC43895 and ATCC43888 (no Stx1 or Stx2 production) were also used.

**Table 1:** Phenotypic and genotypic characteristics of O157:non-H7 and O157:H7 strains used in this study.

Strain	Year	Sor^a^	GUD^b^	O157^c^	*rfbE*	H type^d^	*fliC*-H type^e^	*stx1*	*stx2*	*ehxA*	*eae*	*eae* type^f^	*tir*	*bfpA*	*astA*	*irp2*
**O157:non-H7**																
EC95-42	1995	+	+	+	+	H45	H45	−	−	−	+	α	+	+	+	−
PV51	1996	+	+	+	+	H45	H45	−	−	−	+	α	+	+	+	−
PV52	1996	+	+	+	+	H45	H45	−	−	−	+	α	+	+	+	−
PV405	1997	+	+	+	+	H45	H45	−	−	−	+	α	+	+	+	−
PV00-100	2000	−	+	+	+	H16	H16	−	−	−	−	−	−	−	−	−
PV276	1997	−	+	+	+	NM	H16	−	−	−	−	−	−	−	−	−
PV284	1997	−	+	+	+	NM	H16	−	−	−	−	−	−	−	−	−
PV325	1997	−	+	+	+	NM	H16	−	−	−	−	−	−	−	−	−
PV06-4	2006	−	+	+	+	NM	H16	−	−	−	−	−	−	−	−	−
PV807	1999	+	+	+	+	H16	H16	−	−	−	+	ε	+	−	−	−
PV01-185	2001	+	+	+	+	H16	H16	−	−	−	+	ε	+	−	−	−
PV01-276	2001	+	+	+	+	H16	H16	−	−	−	+	ε	+	−	−	−
PV02-85	2002	+	+	+	+	H16	H16	−	−	−	+	ε	+	−	−	−
PV56	1996	+	+	+	+	H39	H39	−	−	−	+	κ	+	−	−	−
PV57	1996	+	+	+	+	H39	H39	−	−	−	+	κ	+	−	−	−
PV193	1996	+	+	+	+	H39	H39	−	−	−	+	κ	+	−	−	−
PV00-24	2000	+	+	+	+	H43	UT	−	−	−	−	−	−	−	−	−
PV05-43	2005	+	+	+	+	NM	H10	−	−	−	−	−	−	−	−	−
PV01-182	2001	+	+	+	+	UT	UT	−	−	−	−	−	−	−	−	−
PV01-183	2001	+	+	+	+	UT	UT	−	−	−	−	−	−	−	−	−
PV496	1998	+	+	+	+	UT	UT	−	−	−	−	−	−	−	−	+
**O157:H7**																
Sakai	1996	−	−	+	+	H7	H7	+	+	+	+	γ	+	−	−	−
ATCC43895	1982	−	−	+	+	H7	H7	+	+	+	+	γ	+	−	−	−
ATCC43888		−	−	+	+	H7	H7	−	−	+	+	γ	+	−	−	−

a Sor, Sorbitol fermentation.

b GUD, β-glucuronidase activity.

c O-serogroup detected by the *E. coli* O157-specific antibody.

d H-serogroup detected by the *E. coli* H-specific antibodies. NM; non-motile, UT; untypeable.

e genotype detected by the PCR-RFLP assay of the *fliC* gene. UT; untypeable.

f genotype detected by the PCR assay of the *eae* gene.

### Phenotypic Characterization

O serogroups of each strain were determined by agglutination tests with the anti-O157 serum (Denka Seiken Co., Ltd., Tokyo, Japan) according to the manufacturer's instructions. H serogroups were determined using a set of anti-H sera. Sorbitol fermentation (Sor) was detected on Sorbitol MacConkey agar (Nissui Pharmaceutical Co. Ltd, Tokyo, Japan) plates after overnight incubation at 37°C and further confirmed in peptone water containing sorbitol (1%) and Andrade's indicator (1%) after 72 h incubation at 37°C. The b-glucuronidase activity (GUD) of strains was examined with CLIG agar (Kyokuto seiyaku, Tokyo, Japan).

### Genotypic Characterization

Genetic H serotyping was performed by PCR-RFLP analysis of the *fliC* gene (encoding the flagella filament protein) as described previously [10]. The presence of the *rfbE* gene encoding perosamine synthetase, which is essential for O157-antigen biosynthesis was determined by PCR [11]. Furthermore, the following pathotype-associated genes were detected by PCR: *stx1* and *stx2* [12], and *ehxA* [13] associated with EHEC, *eae* [14] and *tir* (encoding translocated intimin receptor) [15] associated with EHEC/EPEC, *bfpA* [16] and EPEC adherence factor (EAF) plasmid specific region [17] associated with typical EPEC, *elt* (encoding heat-labile enterotoxin) and *est* (heat-stable enterotoxin) [18] associated with enterotoxigenic *E. coli* (ETEC), *astA* (encoding heat-stable enterotoxin EAST1) [19], *aggR* (encoding transcriptional activator of aggregative adherence fimbria I expression) [19], and *irp2* (encoding iron-repressible high-molecular-weight protein HMWP2) [20] associated with enteroaggregative *E. coli* (EAEC), *invE* and *ipaH* associated with enteroinvasive *E. coli* (EIEC). All PCRs were performed according to the protocols described previously, except two genes (*invE* and *ipaH*), which were examined using the *Shigella* sp./enteroinvasive *E. coli* (*invE*/*ipaH* genes) PCR Screening Set (TaKaRa Bio Inc., Shiga, Japan). *eae*-positive strains can be classified into several subtypes based on sequence variation within the *eae* gene. Subtyping of the *eae* genes was done by PCR using allele-specific primers (*eae*-alleles: α, β, γ, ε, ζ, ι, η, κ and θ) as described previously [21].

### Sequencing of Seven Housekeeping Genes and *rfbE*

Internal regions of the seven housekeeping genes (*adk*, *fumC*, *gyrB*, *icd*, *mdh*, *purA*, and *recA*) were PCR amplified and sequenced using the primers and protocol specified on the *E. coli* MLST website (http://mlst.ucc.ie/mlst/dbs/Ecoli). The entire coding region of the *rfbE* gene was amplified and sequenced using the primers as follows: rfbE_univ_F (5′-AGCCATTTTGGGTTAACTGTT-3′) and rfbE_univ_R (5′-CCCCACTCGTAAAATCCATCT-3′).

### Evolutionary Analysis

The concatenated sequences of seven housekeeping genes from non-H7 strains were used for multilocus sequence analysis (MLSA). Additionally, the complete genome sequences for the following *E. coli* strains (which are publicly available) were included in the analysis: EHEC strain Sakai (Serogroup O157, Accession number BA000007), EHEC strains 11368 (O26, AP010953), 12009 (O103, 010958) and 11128 (O111, AP010960), EPEC strains E2348/69 (O127, FM180568) and CB9615 (O55, Acc. No. CP001846), ETEC strains E24377A (O139, CP000800) and H10407 (O78, FN649414), EAEC strain 042 (O44, FN554766), Adherent-invasive *E. coli* (AIEC) strain LF82 (O83, CU651637), extraintestinal pathogenic *E. coli* (ExPEC) strains UMN026 (O17, CU928163), IAI39 (O7, CU928164), 536 (O6, CP000247), CFT073 (O6, AE014075), S88 (O45, 928161) and IHE3034 (O18, CP001969), avian pathogenic *E. coli* (APEC) strain (O1, CP000468), commensal *E. coli* strains SE11 (O152, AP009240), SE15 (O150, AP009378), IAI1 (O8, CU928160) and HS (O9, CP000802), and environmental *E. coli* strain SMS-3-5 (O19, CP000970). ECOR strains were also included in the MLSA. Their sequences were obtained from the *E. coli* MLST website: http://mlst.ucc.ie/.

Multiple alignments of sequences were constructed by using the CLUSTAL W program [22] in the MEGA4 software [23], and then neighbor-joining trees were generated by using Tamura-Nei model. A bootstrap test with 1,000 replicates was used to estimate the confidence of the branching patterns of the tree. Rates of non-synonymous (dN) and synonymous (dS) substitutions were estimated using the modified Nei-Gojobori/Jukes-Cantor method in MEGA4 [24]. dN/dS ratio provides a sensitive measure of selective pressure on the protein, with values of dN/dS = 1, >1 and <1 indicating neutral evolution, positive (diversifying) selection and negative (purifying) selection, respectively.

### Sequence Analysis of O157-antigen Biosynthesis Gene Clusters and Their Flanking Regions

The O157-antigen biosynthesis gene cluster and its flanking regions were amplified using three PCR primer pairs as follows: Seg3F (5′-CATAGTCGGTTGGAGTGGCGA T-3′) and Seg3R (5′-TTGCCGGAACGGAGAGAGTAGA-3′) for amplifying the region (18,482 bp in Sakai) covering the entire O157-antigen biosynthesis gene cluster, Seg1F (5′-GATAAAACTCGGGCTCGCCGTG-3′) and Seg2R (5′-TCCGGTACTGGCTATGTAGGCT-3′), and Seg4F (5′-GCCGTTTCAAGTAGTCGGGTTC-3′) and Seg5R (5′-CTTTCCCTTCCAGCCGTTCGTT-3′) for amplifying the upstream (17,205 bp) and downstream (23,954 bp) regions of O157-antigen gene cluster, respectively. Each PCR product was sequenced by the shotgun method. Sequence comparisons were performed using the Sequencher software, ver. 4.9 (Gene Code Corporation, Michigan, USA) and the CLUSTAL W program.

### Data Deposition

The GenBank/EMBL/DDBJ accession numbers for sequences of O157-antigen biosynthesis gene clusters and their flanking regions are EC95-42; AB602249, PV276; AB602250, PV01-185; AB602251, PV57; AB602252, PV00-24; AB602253.

## Results

### Characterization of O157:non-H7 Strains

Basic characteristics of non-H7 strains are shown in Table 1. All strains were reacted with the anti-O157 serum, and genetically confirmed to have the O157-specific *rfbE* gene. Sixteen of 21 strains were motile and their H serogroups determined by H-specific antiserums were as follow: four, H45; five, H16; three, H39; one, H19 and three, untypeable (HUT). Genotypic H serotyping confirmed these results and further revealed that among the five non-motile strains, four were *fliC*-H16 and one was *fliC*-H10. Three strains (PV01-182, PV01-183 and PV496) were typeable neither by serological nor genotypic methods. Although the three H7 strains were Sor− and GUD− phenotypes, all non-H7 strains but five H16 (including NM but *fliC*-H16) were Sor+ and all strains exhibited GUD+.

The presence of virulence-related genes known to be associated with specific pathotypes was examined in each of the non-H7 strains by PCR (Table 1). All of the H45, H16 (Sor+) and H39 strains carried the *eae* and *tir* genes, thus they were classified into EPEC. Depending on the presence or absence of the *eae* gene, the H16 and *fliC*-H16 strains were divided into two groups, termed as H16/*eae*+ and H16/*eae−*. PCR-based intimin typing indicated that the H45, H16/*eae*+ and H39 strains possessed the α, ε and κ subclass intimin, respectively, while H7 strains possessed the γ subclass intimin. In addition, the H45 strains carried the *bfpA* and *astA* genes. Because H45 strains possessed both the *eae* and *bfpA* genes, they were classified into a typical EPEC. No strains possessed the *stx1*, *stx2*, *ehxA*, *elt*, *est*, *aggR*, *irp2*, *invE* or *ipaH* genes, or the specific region of the EAF plasmid (data not shown), except PV496 (HUT) which had *irp2*.

### Phylogenetic Relationships of O157:H7/non-H7 Strains

**Figure 1:**
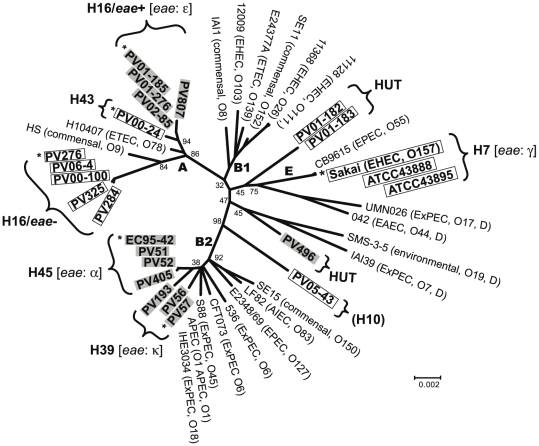
Correlation between evolutionary lineages and distribution of two types of *rfbE* genes among O157:H7/non-H7 stains. 21 fully sequenced *E coli* strains were used as references. The phylogenetic tree was constructed based on concatenated sequences of seven housekeeping genes. *E. coli* phylogroups (A, B1, B2, D and E) were determined by comparing with sequences from the ECOR collection. O157:H7/non-H7 strains carrying “Sakai-type *rfbE*” and “PV01-185-type *rfbE*” are indicated by clear and gray boxes, respectively. Six O157 strains indicated by asterisks were used for the sequence comparisons of O157-antigen gene clusters and their flanking regions.

Based on the concatenated nucleotide sequences (3,423 bp) of seven housekeeping genes, we analyzed the phylogenetic relationships of H7/non-H7 strains. By comparison with the sequences of the ECOR collection strains (data not shown), the H39 and H45 strains belonged to the B2 phylogroup, the H16/*eae*+, H16/*eae−* and H43 strains belonged to the A phylogroup. HUT and *fliC*-H10 strains were unclassified into any of the five major *E. coli* phylogroups. The phylogenetic tree of non-H7 strains with 21 fully sequenced *E. coli* strains expressing various O antigens was constructed (Fig. 1). Non-H7 strains belonged to multiple evolutionary lineages, and all of them were clearly different from that of O157:H7 strains belonging to the E phylogroup. Furthermore, H16/*eae*+ and H16/*eae−* strains formed distinct clusters in the A phylogroup.

### Sequence Analysis of *rfbE*

**Figure 2:**
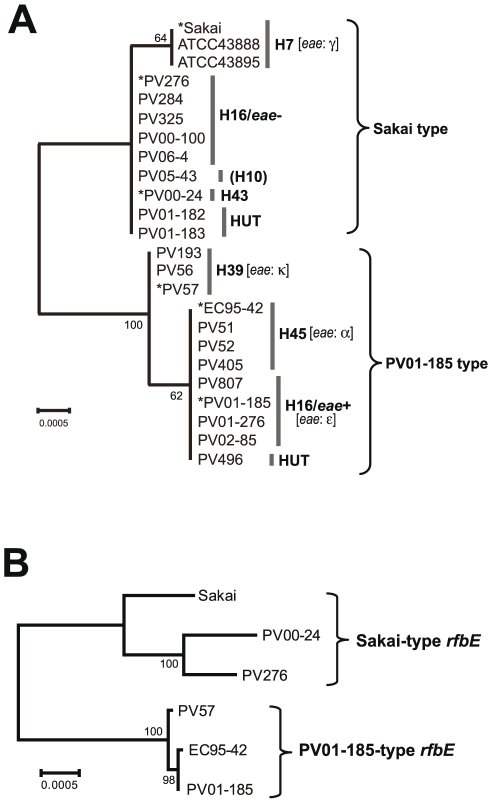
Phylogenetic analysis of the O157-antigen biosynthesis genes. (A) Phylogenetic tree of the *rfbE* from three O157:H7 and 21 O157:non-H7 strains. The six strains indicated by asterisks were used for the sequence comparisons of O157-antigen gene clusters and their flanking regions. (B) Phylogenetic tree of the O157-antigen biosynthesis gene cluster from six O157:H7/non-H7 strains. The tree was constructed based on the concatenated sequences of 12 genes in the O157-antigen gene cluster. Neighbor-joining trees were generated by using Tamura-Nei model. A bootstrap test with 1,000 replicates was used to estimate the confidence of the branching patterns of the tree.

The *rfbE* gene is located in the middle of the O157-antigen biosynthesis gene cluster, and is known to be essential for the synthesis of the O157 antigen. As shown in Fig. 2A, the *rfbE* sequences from 24 H7/non-H7 strains formed two distinct clusters, termed as “Sakai type” and “PV01-185 type”. Sequences from all H16/*eae−*, H43 and *fliC*-H10 strains and two HUT strains were identical and were closely related to those of H7 strains (Sakai type). The PV01-185 type included all H16/*eae*+ (including PV01-185), H45, H39 strains and one HUT strain within one nucleotide difference between H39 strains and others. There were five to seven nucleotide differences between *rfbE* sequences from the “Sakai type” and “PV01-185 type”. Of note is the fact that the distribution of the strains in the two *rfbE* types was inconsistent with the results of the phylogenetic analysis (Fig. 1). For example, although H16/*eae*+ and H16/*eae−* strains were related in the A phylogroup, they carried “PV01-185-type *rfbE*” and “Sakai-type *rfbE*”, respectively.

### Sequence Comparison of the O157-antigen Biosynthesis Gene Clusters and Their Flanking Regions

To gain more information about genetic similarities or differences of the O157-antigen gene clusters as well as their flanking regions, we sequenced about 59 kb of a chromosomal segment containing the O157-antigen gene clusters (13.7 kb) from five representative strains: PV276 (H16/*eae−*) and PV00-24 (H43) for “Sakai-type *rfbE*”, and PV01-185 (H16/*eae*+), EC95-42 (H45) and PV57 (H39) for “PV01-185-type *rfbE*”. Then, fine nucleotide sequence comparison of these strains as well as with EHEC O157:H7 Sakai was performed.

#### O157-antigen biosynthesis gene cluster

**Figure 3:**
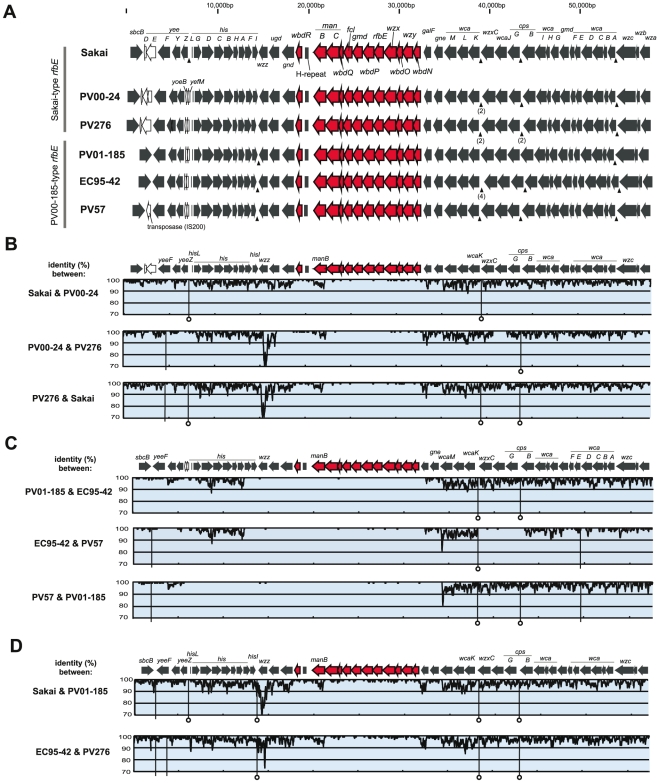
Comparisons of the O157-antigen biosynthesis gene clusters and their flanking regions in six O157 strains. (A) Genetic organization of the O157-antigen gene clusters and their flanking regions. Red arrows indicate orthologs associated with the O157-antigen biosynthesis, and white arrows indicate ORFs that are not conserved in all six strains. Arrowheads indicate insertion sites of REP sequences. (B–D) Pairwise sequence comparisons. (B) Comparisons between O157 strains carrying “Sakai-type *rfbE*”. (C) Comparisons between O157 strains carrying “PV01-185-type *rfbE*”. (D) Comparisons between “Sakai-type *rfbE*” and “PV01-185-type *rfbE*” strains. Sakai is compared with PV01-185, and EC95-42 is compared with PV276. The genetic organization of the O157-antigen gene clusters and their flanking regions are shown in upper panels, and levels of % DNA sequence identity calculated with a 100 bp sliding window and a 10 bp step size are shown in lower panels. The vertical lines indicate regions showing insertion and/or deletion of fragments, and of them, lines with circular heads indi cate indels containing REP sequences.

As shown in Fig. 3A, the gene organization of the O157-antigen gene cluster was identical among six strains, and pairwise sequence comparisons showed that their nucleotide sequences were also highly conserved between the strains (Fig. 3B–D), except for those of *manB* (encoding a phosphomannomutase) of “Sakai-type *rfbE*” strains (Fig. 3B). Concatenated sequences (12,498 bp) of 12 genes from six strains formed two distinct clusters (Fig. 2B). This result is consistent with the result of *rfbE* sequence analysis (Fig. 2A).

ManB might be subjected to selective pressure driven by host-protein interactions, so the nature of the selective pressure was evaluated on the dN/dS ratio among the Sakai-type *rfbE* strains. The average dN/dS ratio of *manB* yielded 0.045 (range: 0.030 to 0.063), and that ratio was not higher than those of the other 11 gene sequences of the O157-antigen gene clusters (dN/dS ratio = 0.282, range: 0.130 to 0.521), indicating that *manB* genes were not influenced by positive selection.

Between strains carrying the “Sakai-type *rfbE*” (Fig. 3B), the region of highly conserved sequence was restricted within the O157-antigen gene cluster. In contrast, between strains carrying the “PV01-185-type *rfbE*” (Fig. 3C), the highly conserved sequence was extended into the flanking regions including part or all of the *his* operon. Although the dN/dS ratio of O157-antigen gene clusters (0.282) showed evidence of purifying selection, that ratio was not lower than those of flanking genes of the O157-antigen gene cluster (*hisG* to *hisC* and *wcaM* to *wcaK*; 0.069 and 0.063, respectively), suggesting that the effect of purifying selection on the O157-antigen gene cluster was lower than those of flanking genes.

#### Regions flanking the O157-antigen biosynthesis gene cluster

The gene organization of regions flanking the O157-antigen gene cluster was almost identical, but Sakai lacked two small genes (corresponding to *yoeB* and *yefM* of *E. coli* K-12) between the *yeeZ* and *hisL* genes, PV57 contained an insertion sequences (IS*200*-like) between the *sbcB* and *yeeF* genes, and three strains carrying the “PV01-185-type *rfbE*” lacked two genes, *yeeD* and *yeeE* (Fig. 3A).

**Figure 4:**
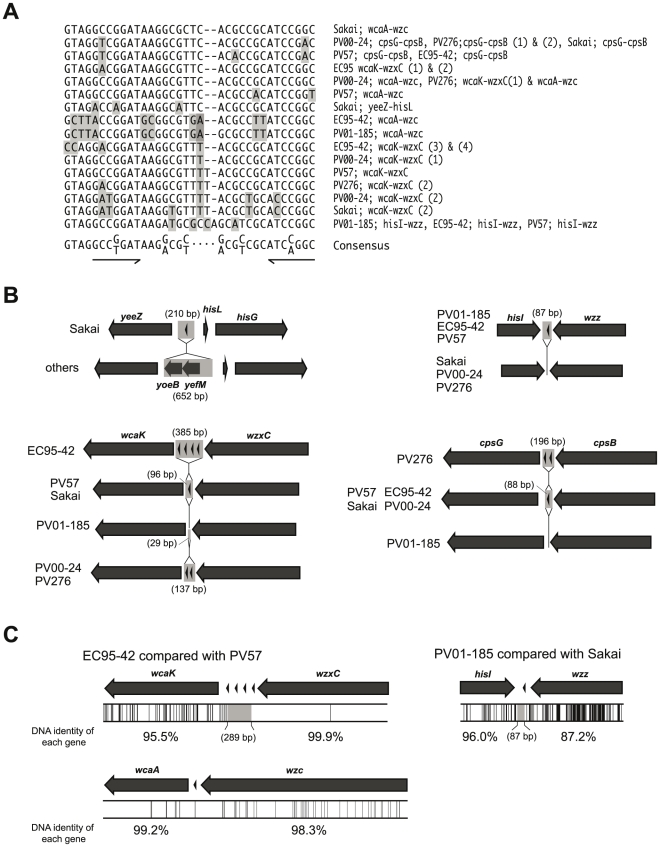
Schematic drawing of REP sequence-containing regions of O157-antigen biosynthesis gene cluster flanking regions. (A) Sequence alignment of the REP sequences located in the O157-antigen gene cluster flanking regions. The consensus sequence is derived from previously published data [40]. The palindromic motif is underlined. The non-consensus sequences were highlighted. (B) Four regions showing insertion and/or deletion of fragments including REP sequence(s); *yeeZ-hisG*, *hisI-wzz wcaK-wzxC* and *cpsG-cpsB* are compared between strains. REP sequences are indicated by arrowheads and gray boxes indicate missing regions on each of the compared strains. (C) The nucleotide sequences from *wcaK* to *wzxC* and from *wcaA* to *wzc* on EC95-42 are compared with those of PV57, and the sequences from *hisI* to *wzz* on PV01-185 are compared with those of Sakai. Locations of SNPs by pairwise sequence comparison are indicated by vertical lines (lower panel).

Pairwise sequence comparisons identified a number of small indels (insertion/deletions) in the O157-antigen gene cluster flanking regions between compared strains, and most of them were located in intergenic regions (Fig. 3B–D). Furthermore, we found several repetitive sequences termed as the repetitive extragenic palindromic (REP) sequences, all of which were located within intergenic regions of *yeeZ*-*hisL*, *hisI*-*wzz*, *wcaK*-*wzxC*, *cpsG*-*cpsB* or *wcaA*-*wzc* (Fig. 3A), and ranged in size from 34 to 36 bp (Fig. 4A). In the region of *wcaA*-*wzc*, all strains conserved a REP sequence (Fig. 3A and Fig. 4C, as an example, between EC95-42 and PV57). In the region of *wcaK*-*wzxC* in EC95-42, four REP sequences were inserted in the same orientation (Fig. 4B). While in the regions of *wcaK*-*wzxC* in EC95-42 and PV276, and *cpsG*-*cpsB* in PV276, two REP sequences were inserted in the same orientation (Fig. 4B). Many indels involved REP sequences (Fig. 4B), and some positions of indels including REP sequences were coincident with junction points of the sequence similarity (Fig. 3B–D and Fig. 4C). Between EC95-42 and PV57, the level of sequence similarity was significantly changed in the intergenic region of *wcaK*-*wzxC* (Fig. 4C), and the *hisI*-*wzz* intergenic region was also observed as a junction point between some strains (as an example, between Sakai and PV01-185, in Fig. 4C).

**Figure 5:**
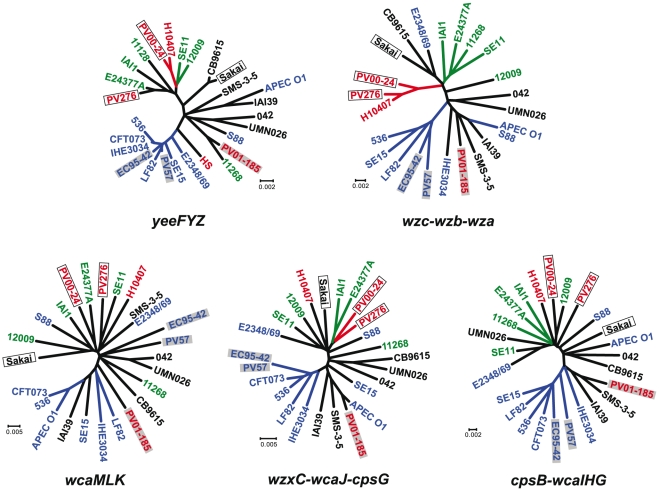
Phylogenetic analyses of the O157-antigen gene cluster-flanking regions. The phylogenetic trees were constructed based on concatenated sequences of *yeeFYZ*, *wcaMLK*, *wzxC-wcaJ-cpsG*, *cpsB-wcaIHG* and *wzc-wzb-wza* by neighbor-joining method using Tamura-Nei model. O157 strains carrying “Sakai-type *rfbE*” and “PV01-185-type *rfbE*” are indicated by clear and gray boxes, respectively. Colors (red, green and blue) indicate their *E. coli* phylogroups (A, B1 and B2), respectively.

To evaluate the evolutionary relationships of regions flanking the O157-antigen gene cluster, concatenated sequences of *yeeZYF* (left hand), *wcaKLM*, *wzxC-wcaJ-cpsG*, *cpsB-wcaGHI* and *wza-wzb-wzc* (right hand) in O157 strains and their homologues in other *E. coli* strains were compared (Fig. 5). Sequences of the *wza-wzb-wzc* formed some clusters and that phylogenetic tree was similar to that of housekeeping genes (Fig. 1), although some sequences (including from PV01-185) did not correlate with their phylogroups. This result indicated that regions covering *wza-wzb-wzc* on PV276, PV00-24, EC95-42 and PV57 were inherited in each lineage during their evolution. Similarly, it was thought that regions covering *yeeZYF* on EC95-42 and PV57 were also inherited in each lineage. In contrast, sequences of *wcaKLM* showed no relationship with their phylogroups, and their sequences from O157 strains did not correlate with their types of the O157-antigen gene cluster.

## Discussions

The O157-antigen gene clusters from O157:H7/non-H7 strains are highly conserved among strains, although they are divided into two distinct types based on nucleotide sequence similarity. The distribution of the two types of O157-antigen gene clusters do not appear to correlate with evolutionary lineages of their strains, which strongly suggests that horizontal transfer of both types of O157-antigen gene clusters has occurred independently among *E. coli* strains.

It is known that EHEC O157:H7 emerged from an O55:H7-like EPEC ancestor by specific events including the acquisition of the O157-antigen biosynthesis gene cluster by horizontal gene transfer [25]. Leopold et al. [26] have performed genome-wide sequence comparison between EHEC O157:H7 and EPEC O55:H7 strains, showing that a large region up to 130 kb including the O-antigen gene cluster, the *his* operon and the colanic acid biosynthesis gene cluster was replaced by the result of the recombination events. The present sequence comparison of O157-antigen gene clusters and their flanking regions between PV01-185-type O157 strains (Fig. 3C) provided evidence that the O-antigen shift has taken place via the horizontal transfer of a large fragment (at least 34 kb, in the case between PV57 and PV01-185) encoding not only the O157-antigen gene cluster but also neighboring genes including the *his* operon. In the case between EC95-42 and PV57 (Fig. 3C, middle graph), the comparable conserved sequences with those of O157-antigen gene clusters were observed in the 4.5 kb region including three genes, *wzxC*, *wcaJ* and *cpsG*, suggesting that the diversified region from *wcaM* to *wcaK* was replaced by another genetic event on EC95-42 and/or PV57.

On the O157-antigen gene cluster, sequences of the *manB* gene had greater divergence than those of the other genes. Samuel *et al*. [27] reported that the *manB* gene originally present in the O157-antigen gene cluster of EHEC O157:H7 has been replaced by an equivalent gene in the colanic acid gene cluster. Indeed, the *cpsG* genes (a homolog of *manB*) in the colonic acid gene clusters of O157 strains have a high level of sequence identity (>95%) with the *manB* genes, and there was no evidence of selective pressure in the *manB* sequences, suggesting that distribution of the *manB* genes of Sakai-type strains can be attributed to gene conversion events with homologous genes within the genome.

The DNA sequences of the *wzz* genes showed significant divergence between some strains. Although not essential for the synthesis and polymerization of O antigen, Wzz regulates the chain length distribution of the O-antigen chain. The length of the O-antigen chain affects various properties of Gram-negative bacteria, including sensitivities to sera [28] and bacteriophages [29], and the function of type III secretion systems [30]. The encoded protein showed only 89.9% amino-acid sequence identity between O157 strains Sakai and PV01-185. These sequence variations in Wzz may affect the length of O157-antigen chains, furthering the virulence potential [30], [31].

Horizontal gene transfer is widely regarded as a major genetic mechanism to shift O serogroups between and within Gram-negative bacterial species, but the mechanism is not well understood. Most evidence obtained by sequence comparisons concluded that the replacement of the incoming fragment was achieved by homologous recombination, because specific sequences that promote specific recombination, such as IS elements and Chi sequences [32] were not found around the possible recombination points. In this study, although these specific sequences were not found in the sequenced regions, except the IS*200*-like element inserted between *sbcB* and *yeeF* on PV57, we noticed the presence of several REP sequences in the regions flanking the O157-antigen gene clusters. REP sequences are highly repetitive sequences found in the chromosome of *E. coli* and other bacteria [33]. The presence of REP sequences has been related to several functions, such as mRNA stabilization [34] and transcription control [35]. Additionally, REP-related sequences are known as binding sites for DNA polymerase I [36], DNA gyrase [37], and integration host factor (IHF) [38], and hotspots of IS element integration [39]. Although it is not clear whether REP sequences are associated with the O157-antigen shifts, it is reasonable to suggest that some genomic rearrangements of chromosomal regions flanked by the O157-antigen gene clusters may have involved the REP sequences. In this study, we could find only a few cases where REP sequences were appeared to involve with the horizontal gene transfer. A genomewide comparative analysis will help to better understand the function of the REP sequence involved in the horizontal gene transfer.

In summary, the present study showed that O157:non-H7 strains belong to multiple evolutionary lineages distinct from EHEC O157:H7 strains, regardless of the *eae*-positive or -negative strains. Although all O157 strains possessed highly conserved O157-antigen gene clusters, these clusters were divided into two distinct types at the nucleotide sequence level, and surprisingly, their distribution did not follow the evolutionary lineages of the strains. From these results, we conclude that horizontal transfer of the two types of O157-antigen gene clusters induced the emergence of multiple O157 lineages within *E. coli*. Our results provide novel information regarding the distribution of O157-serogroup strains in *E. coli*. Additionally, we speculate that REP sequences in the regions flanking the O-antigen gene clusters may involve one of the driving forces for exchange and evolution of O-antigen loci. To better understand the genetic mechanism(s) generating wide variety of O serogroups, we need to know more about whether REP sequences is involved in O-antigen shifts in other *E. coli* O serogroups and also in other Gram-negative bacterial species.
